# The effects of body mass index on outcomes for patients undergoing surgical aortic valve replacement

**DOI:** 10.1186/s12872-020-01528-8

**Published:** 2020-05-29

**Authors:** Keir Forgie, Sabin J. Bozso, Yongzhe Hong, Colleen M. Norris, Abdullah Ishaque, Richdeep S. Gill, Darren H. Freed, Michael C. Moon, Jayan Nagendran, Jeevan Nagendran

**Affiliations:** 1 Division of Cardiac Surgery, Department of Surgery, University of Alberta, Edmonton, Alberta Canada; 2 Mazankowski Alberta Heart Institute, University of Alberta, Edmonton, AB Canada; 3 University of Alberta, Medical School, Edmonton, Alberta Canada; 4 Division of General Surgery, University of Calgary, Calgary, Alberta Canada; 5 Cardiac Surgeon, Minimally Invasive Valve Surgery, University of Alberta, Edmonton, Canada

**Keywords:** Aortic valve replacement, Heart (incl related subjects), Obesity (incl related subjects), Outcomes (incl mortality, Morbidity, Survival, Etc.)

## Abstract

**Background:**

Most of the studies of obesity and postoperative outcome have looked predominantly at coronary artery bypass grafting with fewer focused on valvular disease. The purpose of this study was to compare the outcomes of patients undergoing aortic valve replacement stratified by body mass index (BMI, kg/m^2).

**Methods:**

The Alberta Provincial Project for Outcome Assessment in Coronary Heart Disease registry captured 4780 aortic valve replacements in Alberta, Canada from January 2004 to December 2018. All recipients were stratified by BMI into five groups (BMI: < 20, 20–24.9, 25–29.9, 30–34.9, and > = 35). Log-rank test and Cox regression were used to examine the crude and adjusted survival differences.

**Results:**

Intra-operative clamp time and pump time were similar among the five groups. Significant statistical differences between groups existed for the incidence of isolated AVR, AVR and CABG, hemorrhage, septic infection, and deep sternal infection (*p* < 0.05). While there was no significant statistical difference in the mortality rate across the BMI groups, the underweight AVR patients (BMI < 20) were associated with increased hazard ratio (1.519; 95% confidence interval: 1.028–2.245) with regards to all-cause mortality at the longest follow-up compared with normal weight patients.

**Conclusion:**

Overweight and obese patients should be considered as readily for AVR as normal BMI patients.

## Background

Obesity rates in cardiac surgery are increasing and outcomes research within this population have led to the discovery of “the obesity paradox” [1]. Within the general population, obesity is a risk factor for cardiovascular disease and mortality [2]. Obesity is objectively defined in the epidemiology literature via body mass index (BMI), which is the ratio of mass to height in kilograms/meter^2^ [3]. Intuitively, obesity should be associated with an increased operative risk in cardiac surgery and is often perceived that way [4, 5]; however, that is not what research suggests and obesity may in fact be protective [1, 6]. The counterintuitive finding of reduced mortality in obese patients post cardiac surgery is known as the obesity paradox.

Many studies support the notion of an obesity paradox [7–10]; however, most of the published literature was based on observational data, limited by small sample sizes, and inadequate long-term follow-up, thereby restricting meaningful conclusions. Furthermore, previous studies focused predominantly on BMI in relation to coronary artery bypass grafting with fewer focused on valvular disease [8, 9, 11–14].

The aim of this study was to better understand the impact of BMI on outcomes of patients undergoing aortic valve replacement (AVR). We performed a retrospective study that compared AVR outcomes stratified across a broad range of BMI using a database that captures all cardiac catheterization in Alberta, Canada over a 15-year period.

## Methods

Aortic valve replacement patients from 2004 to 2018 in the Alberta Provincial Project for Outcome Assessment in Coronary Heart Disease (APPROACH) registry were included in this study. Exclusion Criteria included: less than 18 years of age, emergent surgery, and transplant recipients. The APPROACH project is a province-wide inception cohort of all adult Alberta residents undergoing cardiac catheterization. The APPROACH registry contains detailed clinical data collected at catheterization, percutaneous coronary intervention, surgery, and one, three and five year follow-up. Finally, there is a quarterly merge with the Alberta Bureau of Vital Statistics to provide mortality data. The human research ethics office at the University of Alberta granted ethics approval prior to accessing the database. All procedures performed in studies involving human participants were in accordance with the ethical standards of the institutional and/or national research committee and with the 1964 Helsinki declaration and its later amendments or comparable ethical standards. For this type of study, formal consent was not required. Patients were grouped based on their preoperative BMI into five groups: BMI < 20 (underweight), BMI 20–24.9 (normal weight), 25–29.9 (overweight), and 30–34.9 (obese), and > 35 (morbidly obese). In order to prevent having too few underweight patients to achieve statistical significance, this study used a BMI < 20 for underweight patients. Relevant preoperative characteristics were obtained for all patients. Preoperative known risk factors that could impact the outcome of AVR were also identified and included. Similarly, the peri-operative variables and risk factors, including length of surgery, ischemic time and cross-clamp data, were collected along with post-operative complications. Primary outcomes were mortality rate at 30-day, 1 year, and 5 years. Secondary outcomes were rehospitalization for MI, rehospitalization for stroke, and redo AVR.

### Statistical analysis

Continuous variables were summarized as mean ± SD or as median (interquartile range) if not normally distributed and categorical variables as frequency (percent). Continuous variables were compared using Student’s t-test or Mann-Whitney U test in cases of non-normal distribution. Categorical variables were compared with Chi-square test or the Fisher exact test as appropriate. Log-rank test was used to compare the unadjusted primary and secondary outcomes between BMI groups. Multivariable logistic regression was performed to compute the adjusted odds ratios for superficial sternal wound infection. Cox proportional hazards regression models were used to determine the association between BMI and the primary and secondary outcomes, after adjusting for all the variables included in Table 1. Then, a sensitivity analysis adding bypass time, cross-clamp time, hemorrhage, CVICU duration, concomitant CABG, MV intervention (replacement or repair) and TV intervention as covariates in the previous Cox regression was explored. The proportional hazard assumption was tested by adding an interaction term of BMI and time to the full model, for which *p* value less than 0.05 indicated violation of assumption. No violations were found in all the Cox regression models. Pre-specified subgroup analyses by sex and in patients with aortic stenosis and post hoc analysis in patients with severe aortic insufficiency were also performed using Cox regression analysis. Adjusted survival curves were generated and Hazard ratios (95% CIs) referenced to normal weight patients were reported for each Cox regression model. Statistical analysis was performed using the SPSS software version 24 (SPSS, Chicago, Illinois). A *p* value < 0.05 was considered statistical significance.

**Table 1:** Preoperative Characteristics of study participants stratified by BMI

Independent Variables	BMI < 20 (*N* = 108)	BMI 20–24.9 (*N* = 972)	BMI 25–29.9 (*N* = 1721)	BMI 30–34.9 (*N* = 1199)	BMI ≥ 35 (*N* = 780)	*P* value
Age (years)	60.1 ± 20.3	64.6 ± 16.7	66.2 ± 14.0	66.3 ± 12.2	64.3 ± 11.4	< 0.001
Male (%)	53(49.1)	647(66.6)	1304(75.8)	884(73.7)	476(61.0)	< 0.001
Chronic Lung Disease (%)	38(35.2)	309(31.8)	510(29.6)	379(31.6)	283(36.3)	0.021
Chronic Renal Insufficiency (%)	10(9.3)	94(9.7)	176(10.2)	135(11.3)	91(11.7)	0.586
Hypertension (%)	60(55.6)	576(59.3)	1193(69.3)	912(76.1)	645(82.7)	< 0.001
Hyperlipidemia (%)	60(55.6)	646(66.5)	1272(73.9)	944(78.7)	612(78.5)	< 0.001
Diabetes(%)	10(9.3)	134(13.8)	320(18.6)	372(31.0)	315(40.4)	< 0.001
Current smoker(%)	20(18.5)	147(15.1)	528(30.7)	184(15.3)	104(13.3)	0.012
Prior MI (%)	1(0.9)	24(2.5)	80(4.6)	64(5.3)	41(5.3)	0.003
Prior PCI (%)	7(6.5)	47(4.8)	130(7.6)	121(10.1)	70(9.0)	< 0.001
Prior CABG (%)	4(3.7)	34(3.5)	71(4.1)	66(5.5)	23(2.9)	0.050
PVD (%)	2(1.9)	49(5.0)	68(4.0)	53(4.4)	19(2.4)	0.048
CVD (%)	7(6.5)	106(10.9)	180(10.5)	99(8.3)	65(8.3)	0.074
Dialysis (%)	4(3.7)	19(2.0)	14(0.8)	12(1.0)	9(1.2)	0.012
Malignancy (%)	2(1.9)	34(3.5)	61(3.5)	52(4.3)	28(3.6)	0.617
Liver Disease (%)	1(0.9)	10(1.0)	20(1.2)	6(0.5)	6(0.8)	0.433
GI disease (%)	20(18.5)	147(15.1)	281(16.3)	184(15.3)	104(13.3)	0.335
**Ejection Fraction**						0.094
< 20%	2(1.9)	13(1.3)	19(1.1)	9(0.8)	8(1.0)	
20–34%	10(9.3)	88(9.1)	134(7.8)	91(7.6)	54(6.9)	
35–50%	28(25.9)	196(20.2)	354(20.6)	213(17.8)	127(16.3)	
> 50%	63(58.3)	634(65.2)	1140(66.2)	847(70.6)	562(72.1)	
Unavailable	5(4.6)	41(4.2)	74(4.3)	39(3.3)	29(3.7)	

*Abbreviations*: *BMI* body mass index, *CABG* coronary artery bypass graft, *CVD* cerebrovascular disease, *GI* gastrointestinal, *MI* myocardial infarction, *N* number, *PCI* percutaneous coronary intervention, *PVD* peripheral vascular disease, *SD* standard deviation

## Results

### Baseline demographics

From 1 January 2004 to 31 December 2018, a total of 4780 consecutive patients who underwent aortic valve replacement were stratified by BMI into five categories: underweight (BMI < 20, *n* = 108, 2.3%), normal weight (BMI 20–24.9, *n* = 972, 20.3%), overweight (BMI = 25–29.9, *n* = 1721, 36.0%), obese (BMI = 30–34.9, *n* = 1199, 25.1%), and morbidly obese (BMI > 35, *n* = 780, 16.3%). Baseline characteristics and significance are summarized in Table 1. Table 1 demonstrates various preoperative characteristics that were not significantly different among the five groups. These risk factors included chronic renal insufficiency, cerebral vascular disease (CVD), malignancy, liver disease, and gastrointestinal (GI) disease.

Table 1 also demonstrates various preoperative characteristics that were significantly different among the five groups. These risk factors included age, sex, chronic lung disease, hypertension, hyperlipidemia, diabetes, current smoking status, prior MI, prior PCI, prior CABG, peripheral vascular disease (PVD), and dialysis (*p* < 0.05).

### Perioperative analysis and post-AVR survival

Intra-operative and post-operative characteristics are summarized in Table 2. Intra-operative clamp time and pump time were similar among the five groups, and there was no significant difference in incidence of surgical implantation of mechanical versus bioprosthetic valves. Post-operatively, there was no significant difference in CVICU length of stay (4.7 ± 6.8, 3.9 ± 10.2, 3.4 ± 8.0, 3.4 ± 4.8, 4.2 ± 6.8 days; underweight, normal weight, overweight, obese and morbidly obese respectively, *p* = 0.055) or ventilator time (1.3 ± 2.2, 1.3 ± 12.4, 1.1 ± 11.2, 1.2 ± 11.4, 1.2 ± 4.8 days; underweight, normal weight, overweight, obese and morbidly obese respectively, *p* = 0.999). Significant statistical differences between groups did exist for the incidence of isolated AVR, AVR with CABG, AVR with MVR, AVR with TV repair, hemorrhage, septic infection, superficial and deep sternal infection (*p* < 0.05). Secondary outcomes showed, no significant statistical differences between groups for the incidence of rehospitalization for MI, stroke, or re-do AVR (Tables 3 and 4).

**Table 2:** Intra−/postoperative characteristics of study participants stratified by BMI

Independent Variables	BMI < 20 (N = 108)	BMI 20–24.9 (N = 972)	BMI 25–29.9 (N = 1721)	BMI 30–34.9 (N = 1199)	BMI ≥ 35 (N = 780)	*P* value
Isolated AVR (%)	32(29.6)	286(29.4)	506(29.4)	373(31.1)	280(35.9)	0.017
AVR + CABG (%)	30(27.8)	326(33.5)	628(36.5)	446(37.2)	221(28.3)	< 0.001
AVR + MVR(%)	11(10.2)	83(8.5)	101(5.9)	38(3.2)	39(5.0)	< 0.001
AVR + TV repair (%)	8(7.4)	56(5.8)	54(3.1)	28(2.3)	29(3.7)	< 0.001
**Valvular type**						0.370
Mechanical Valve (%)	19(17.6)	133(13.7)	224(13.0)	141(11.8)	106(13.6)	
Bioprosthetic Valve (%)	89(82.4)	839(86.3)	1497(87.0)	1058(88.2)	674(86.4)	
Clamp time (min)	124.4 ± 58.8	119.9 ± 50.4	117.8 ± 50.5	117.1 ± 47.5	117.4 ± 50.3	0.522
Pump time (min)	164.0 ± 80.1	151.9 ± 59.8	150.0 ± 59.3	150.2 ± 59.1	150.0 ± 63.4	0.237
***Postoperative characteristics***						
Hemorrhage (%)	8(7.4)	31(3.2)	32(1.9)	17(1.4)	17(2.2)	< 0.001
Infection						
Septic Infection (%)	5(4.6)	22(2.3)	24(1.4)	12(1.0)	20(2.6)	0.005
Superficial sternal wound infection	3(2.8)	7(0.7)	32(1.9)	30(2.5)	30(3.8)	< 0.001
Sternal wound cellulitis	0(0.0)	0(0.0)	1(0.1)	0(0.0)	0(0.0)	0.777
Deep Sternal Infection (%)	1(0.9)	5(0.5)	3(0.2)	1(0.1)	7(0.9)	0.016
Sternal wound dehiscence (sterile)	0(0.0)	0(0.0)	1(0.1)	1(0.1)	0(0.0)	0.847
Ventilator (days)	1.3 ± 2.2	1.3 ± 12.4	1.1 ± 11.2	1.2 ± 11.4	1.2 ± 4.8	0.999
CVICU (days)	4.7 ± 6.8	3.9 ± 10.2	3.4 ± 8.0	3.4 ± 4.8	4.2 ± 6.8	0.055

*Abbreviations*: *AVR* aortic valve replacement, *MVR* mitral valve replacement, *TV* tricuspid valve, *CABG* coronary artery bypass graft, *BMI* body mass index, *min* minutes, *N* number, *ICU* intensive care unit

**Table 3:** Primary and secondary outcomes for study participants stratified by BMI

	BMI < 20 (N = 108)	BMI 20–24.9 (N = 972)	BMI 25–29.9 (N = 1721)	BMI 30–34.9 (N = 1199)	BMI ≥ 35 (N = 780)	*P* value
**Primary Outcome**						
*Death at 30 days*	2(1.9)	15(1.5)	23(1.3)	10(0.8)	15(1.9)	0.313
*Death at 1 year*	9(8.3)	46(4.7)	81(4.7)	47(3.9)	38(4.9)	0.300
*Death at 5 year*	19(17.6)	126(13.0)	229(13.3)	150(12.5)	100(12.8)	0.725
Death at longest follow-up	29(26.9)	229(23.6)	377(21.9)	237(19.8)	159(20.4)	0.519
**Secondary outcome**						
Hospitalization for Myocardial Infarction	2(1.9)	41(4.2)	69(4.0)	69(5.8)	28(3.6)	0.061
Hospitalization for Stroke	7(6.5)	64(6.6)	136(7.9)	84(7.0)	41(5.3)	0.440
Redo AVR	2(1.9)	18(1.9)	28(1.6)	22(1.8)	20(2.6)	0.151

**Table 4:** Impact of weight categories on the outcomes of patients undergone AVR, multivariable Cox regression analysis

	Hazard ratio	95% CI		***P*** value
		Lower	Upper	
**All-cause mortality at longest follow up**				
20–25 (Normal BMI)	Reference			
< 20	1.519	1.028	2.245	0.036
25–30	0.995	0.843	1.175	0.955
30–35	0.906	0.751	1.094	0.304
> =35	1.090	0.879	1.351	0.432
**Rehospitalization for MI**				
20–25 (Normal BMI)	Reference			
< 20	0.491	0.118	2.042	0.328
25–30	0.935	0.632	1.382	0.735
30–35	1.267	0.849	1.890	0.247
> =35	0.829	0.501	1.372	0.465
**Rehospitalization for Stroke**				
20–25 (Normal BMI)	Reference			
< 20	1.174	0.535	2.574	0.690
25–30	1.184	0.877	1.598	0.270
30–35	1.018	0.729	1.421	0.919
> =35	0.809	0.537	1.218	0.310
**Redo AVR**				
20–25 (Normal BMI)	Reference			
< 20	0.661	0.149	2.930	0.586
25–30	0.831	0.455	1.519	0.548
30–35	1.097	0.578	2.082	0.778
> =35	1.593	0.799	3.173	0.186

The unadjusted mortality rate at 30-day, 1 year, 5 years, and longest follow up are shown in Table 3. The median follow-up time and its interquartile range for the entire population was 5.11(6.03) years. There was no statistically significant difference in mortality rate across the five groups. Table 4 shows the adjusted impact of weight categories on the primary and secondary outcomes of patients using multivariable cox regression with normal BMI as a reference. The hazard ratio for the BMI < 20 group was statistically significant compared to the reference group for all-cause mortality (HR 1.52, 95% Confidence Interval (CI) 1.03–2.25). There was no statistically significant difference between any of the groups when compared to the reference group (normal weight patients) for rehospitalization for MI, rehospitalization for stroke, and redo AVR (Table 4).

Table 5 shows the results of an adjusted logistic regression on superficial sternal wound infections. Compared to the reference population of normal BMI, all cohorts demonstrated statistically significant differences in the incidence of superficial wound infection (OR 4.39, 1.10–17.54; 2.58, 1.13–5.90; 3.55, 1.53–8.23; 5.40, 2.29–12.71; underweight, overweight, obese and morbidly obese groups, respectively).

**Table 5:** Logistic regression on superficial sternal wound infection

	Odds ratios	95% CI		***P*** value
		Lower	Upper	
Superficial sternal wound infection				
20–25 (Normal BMI)	Reference			
< 20	4.387	1.097	17.540	0.036
25–30	2.578	1.126	5.901	0.025
30–35	3.552	1.533	8.232	0.003
> =35	5.397	2.293	12.706	< 0.001

**Fig. 1:**
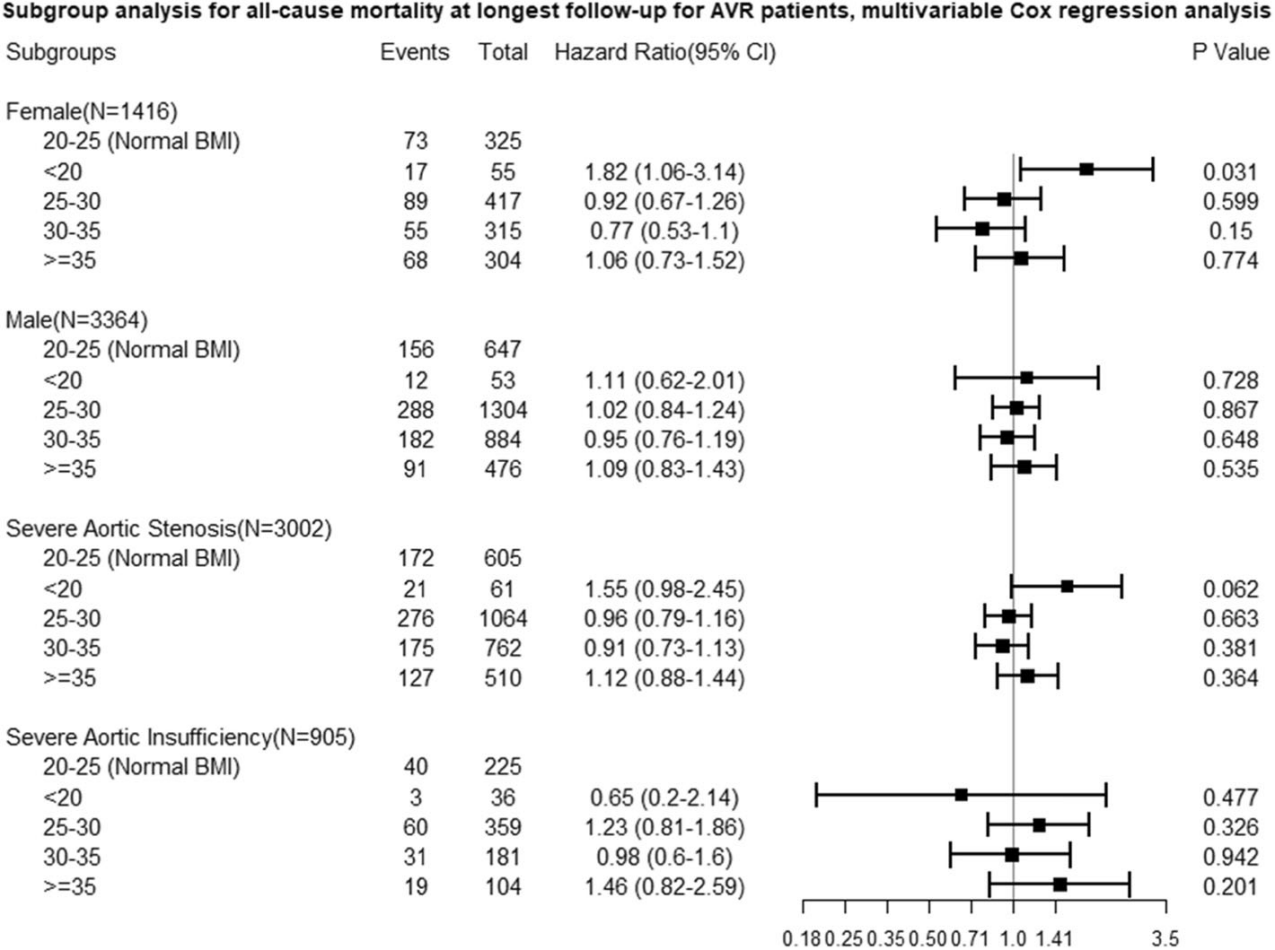
Subgroup analysis for all-cause mortality at longest follow-up for AVR patients, multivariable Cox regression analysis

**Fig. 2:**
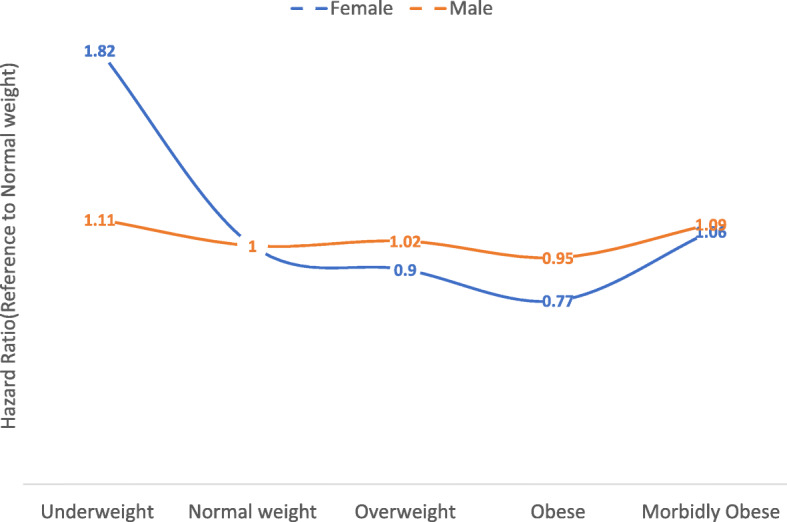
Spline curve of Hazard ratio for all-cause mortality at longest follow-up against BMI groups (Reference to Normal weight)

Table 6 and Fig. 1 show a sub-group analysis for the BMI groups for the primary outcome of mortality and longest follow-up. In terms of sex, the HR for all-cause mortality at longest follow up among underweight females (BMI < 20) remained increased compared to the normal BMI reference group; however, the HR for underweight males did not (Fig. 2). The HR for the surgical indication of severe aortic stenosis was increased at 1.548 for the underweight group; however, it did not reach statistical significance at *p* = 0.062. We found no evidence of significant differences in survival across the BMI groups in the severe aortic insufficiency population.

**Table 6:** Subgroup analysis for all-cause mortality at longest follow-up for AVR patients, multivariable Cox regression analysis

	Events	No. at risk	Hazard ratio	95% CI		***P*** value
				Lower	Upper	
**SUBGROUP ANALYSIS**						
**Female (*N*** **= 1416)**						
20–25 (Normal BMI)	73	325	Reference			
< 20	17	55	1.820	1.056	3.138	0.031
25–30	89	417	0.919	0.670	1.260	0.599
30–35	55	315	0.765	0.532	1.101	0.150
> =35	68	304	1.055	0.733	1.517	0.774
**Male (*N*** **= 3364)**						
20–25 (Normal BMI)	156	647	Reference			
< 20	12	53	1.111	0.615	2.008	0.728
25–30	288	1304	1.017	0.835	1.239	0.867
30–35	182	884	0.950	0.761	1.185	0.648
> =35	91	476	1.090	0.830	1.431	0.535
**Severe Aortic stenosis (*N*** **= 3002)**						
20–25 (Normal BMI)	172	605	Reference			
< 20	21	61	1.548	0.978	2.449	0.062
25–30	276	1064	0.958	0.789	1.163	0.663
30–35	175	762	0.906	0.727	1.130	0.381
> =35	127	510	1.122	0.875	1.440	0.364
**Severe Aortic Insufficiency(*N*** **= 905)**						
20–25 (Normal BMI)	40	225	Reference			
< 20	3	36	0.649	0.197	2.138	0.477
25–30	60	359	1.230	0.813	1.861	0.326
30–35	31	181	0.982	0.602	1.602	0.942
> =35	19	104	1.456	0.818	2.590	0.201

## Discussion

**Fig. 3:**
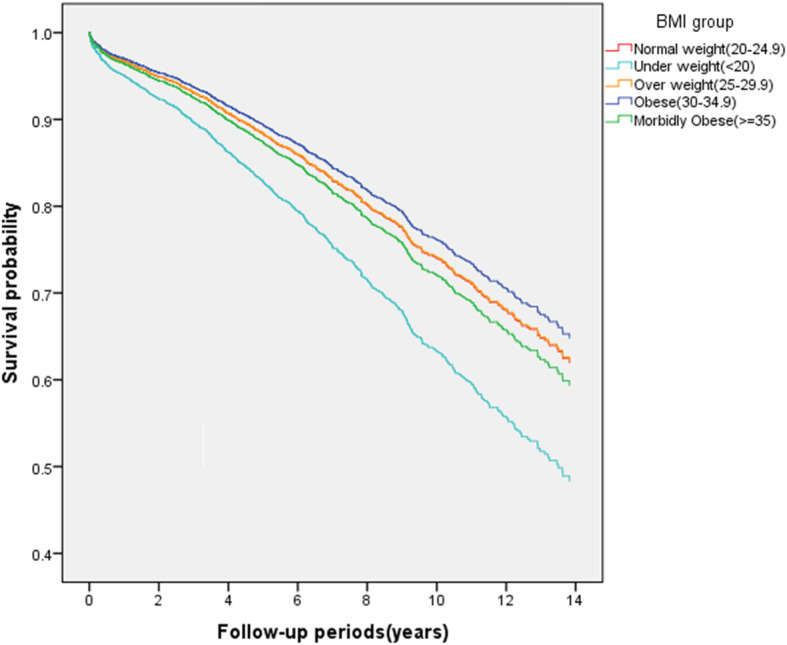
Adjusted survival curve for all cause mortality at longest available follow-up. (Normal weight and overweight group overlapped)

Although previous studies have established that obesity is associated with improved survival following cardiac surgery [15], the present work expands upon this data across a range of BMIs in AVR with or without concomitant CABG. The results show that obese patients (BMI 30–34.9) and morbidly obese patients (BMI > 35) do not experience decreased long-term survival post AVR compared to normal weight (BMI 20–24.9) and overweight (BMI 25–29.9) cardiac surgery patients. In fact, underweight patients (BMI < 20) compared to all other BMI groups have significantly increased all-cause mortality at long-term follow-up (Fig. 3).

These results are consistent with those of several observational studies examining AVR outcomes in patients with relation to BMI. Florath [16] showed that patients with BMI > 30, 35, and 40 did not experience increased risk of 30 day or 6-month mortality after AVR. Interestingly, Florath did show that a low BMI (< 24) independently predicted an increased mortality at 30 days and 6 months post AVR. This finding is similar to our own in that low BMI (< 20) is negatively associated with survival. Vaduganathan [17] reported similar results that overweight (BMI 25–29.9) and obese (BMI 30–60) patients were at a lower hazard of long-term all-cause mortality compared to normal BMI (18.5–24.9). Also, patients who were underweight (BMI 11.5–18.4) were at a greater adjusted risk of long-term mortality post AVR compared to normal weight patients. Although there are slight variations in the BMI cut-offs per group, the overall findings between these studies and our own are congruent. BMIs > 30 are not associated with increased mortality and perhaps are protective, while BMI approximately less than 20 is associated with increased risk of all-cause mortality. We performed a sensitivity analysis (Table S1) by including the bypass time, cross-clamp time, hemorrhage, length of CVICU stay, concomitant CABG, MV intervention and TV intervention in the Cox regression for long-term survival. We failed to find evidence that there is a difference in the long-term survival among the 5 BMI groups. (Underweight: HR 1.304, 95%CI 0.856–1.987; Overweight: HR 1.121, 95%CI 0.940–1.336; Obese: HR 1.048, 95%CI 0.860–1.276; Morbidly Obese: HR 1.238, 95%CI 0.986–1.555) This may indicate that disease severity could be an intermediate factor between underweight and higher risk of death. Since underweight is often considered a marker for cachexia, frailty and sarcopenia, underweight patients are more susceptible to postoperative complications and delayed recovery, which could partly explain the underlying mechanism of higher mortality in the underweight group [18]. As BMI is a modifiable risk factor, studies on whether nutritional support could improve patients’ survival are needed.

Furthermore, significant preoperative, intraoperative, and postoperative characteristic differences in the underweight group increase their risk of mortality, independently from their low BMI, including having a lower LVEF, increased CVICU length of stay, increased rate of dialysis and an increased proportion undergoing concomitant mitral and tricuspid valve replacements. As well, compared to the reference group, the underweight group had a greater proportion of patients with an EF between 35 and 50%. The average number of days in CVICU was greater for the underweight group; however, no significant difference was found across the five groups. A significant difference did exist between the five groups with regards to the proportion of patients requiring dialysis, with the greatest proportion in the underweight group. This finding corresponds to the greater length of stay in CVICU as well as increased mortality rate. Furthermore, the underweight group had a greater proportion of patients undergo concomitant valve surgeries, and these more complex procedures will also correspond to increased CVICU length of stay and decreased long-term survival outcomes. Taken together, there are several factors in the underweight cohort that correlate with a survival disadvantage and the precise effect of each variable on mortality will need further research. It is noted from this study that a BMI less than 20 is an independent risk factor for long-term survival.

Our study has several additional interesting findings that expand on the current literature. Our subgroup analysis demonstrated that females in the underweight group were at the greatest risk of all-cause mortality. This suggests that there is increased vulnerability associated with female cardiac surgery patients who present as underweight for surgery. Studies have previously shown that females represent a higher risk group for SAVR compared to males, with worse survival outcomes, yet other studies demonstrated conflicting results with regards to the differential impact of sex outcomes following SAVR [19]. Further research into sex-related outcomes in SAVR is required. Another interesting finding was reflected in Table 6, which showed that all groups other than normal BMI experienced statistically significant increased incidence of superficial sternal wound infections post-op. This suggests that the healing properties of the tissues are negatively affected by BMIs outside the normal range. Also, the severity of increased risk with regards to superficial sternal wound infections appears to be the worst for the cohort of BMI > 35. Given this, patients with BMI > 35 should have additional wound assessment post-operatively to guard against wound infection and perhaps should receive longer courses of antibiotics than usual.

This study is not without limitations. We used retrospectively collected data on adult cardiac surgery patients from two institutions in one Canadian province, with long term (2004–2018) follow up through linkage of electronic mortality records; however, our study is limited by its observational design, which does not allow us to explore causal relationships, only comment on noteworthy associations. Of note, we did not have access to patients’ body surface area (BSA) measurements; therefore, we were unable to assess for patient-prosthesis mismatch (PPM) in each of the BMI categories. Furthermore, we did not have complete data with regards to the functional class of all patients; therefore, this information has not been included in the analysis. Also, we have not performed STS risk calculations on the included patients; however, we do acknowledge that STS score is an important aspect of aortic valve procedures. Our main reason behind this is the possibility that adding a risk score as a covariate may over-adjust the effect and bias towards the null. Lastly, 156 (3.3%) patients from our study population were diagnosed with native aortic valve infectious endocarditis as the indication for AVR. This is a higher risk surgical population compared to elective AS or AI. That said, the purpose of this study was to examine the outcomes of AVR in patients across a range of BMI with long term follow-up to aid in clinical decision-making and risk stratification as well as further elucidate the obesity paradox. To this end, we have further supported the notion of an obesity paradox and highlighted the increased risk associated with low BMI cardiac surgery patients.

## Conclusions

We have shown that obesity and morbid obesity is not associated with reduced survival post AVR and AVR with CABG, and this should be considered when assessing patients. However, underweight patients (BMI < 20) do experience decreased long-term survival compared with all other BMI groups, and should therefore be considered higher risk surgical candidates. This particularly finding of high risk with low BMI has received comparatively less focus in the available literature and thus represents a gap in the research on cardiac surgical outcomes.

## Supplementary information

**Additional file 1: Table S1.** Sensitivity analysis of the impact of weight categories on the all-cause mortality at longest follow up for patients undergone AVR, multivariable Cox regression analysis (Bypass time, cross-clamp time, hemorrhage, length of CVICU stay, concomitant CABG, MV intervention (replacement or repair) and TV intervention were included as covariates in the Cox regression model in addition to the variables in Table 1).

## Data Availability

All data were collected from a combined cardiology and cardiac surgery database. This database, the Alberta Provincial Project for Outcome Assessment in Coronary Heart Disease (APPROACH), is a prospective data collection registry that collects real-time data from three hospital sites, beginning at the patient’s referral for car- diac catheterization. The APPROACH database has captured information on all patients undergoing cardiac catheterization in Alberta, Canada, a province of approximately 4 million people. All data analyzed during this study are included in this published article and its supplementary information files.

## Abbreviations

APPROACH: Alberta provincial project for outcome assessment in coronary heart disease
AI: Aortic insufficiency
AS: Aortic stenosis
AVR: Aortic valve replacement
BMI: Body mass index (kg/m^2)
BSA: Body surface area
CABG: Coronary artery bypass grafting
CI: Confidence interval
CVD: Cerebrovascular disease
CVICU: Cardiovascular intensive care unit
GI: Gastrointestinal disease
HR: Hazard ratio
MI: Myocardial infarction
MV: Mitral valve
MVR: Mitral valve replacement or repair
N: Number
OR: Odds ratio
PCI: Percutaneous coronary intervention
PPM: Patient-prosthesis mismatch
PVD: Peripheral vascular disease
SAVR: Surgical aortic valve replacement
SD: Standard deviation
STS: Society of Thoracic Surgeons
TV: Tricuspid valve
