# Supplementary material for: The effects of body mass index on outcomes for patients undergoing surgical aortic valve replacement
Source: BMC Cardiovasc Disord. 2020 May 29;20:255. doi: 10.1186/s12872-020-01528-8 (PMC7256925; doi:10.1186/s12872-020-01528-8)
Supplement: Supplementary file 1 — Additional file 1: Table S1. Sensitivity analysis of the impact of weight categories on the all-cause mortality at longest follow up for patients undergone AVR, multivariable Cox regression analysis (Bypass time, cross-clamp time, hemorrhage, length of CVICU stay, concomitant CABG, MV intervention (replacement or repair) and TV intervention were included as covariates in the Cox regression model in addition to the variables in Table 1). [file 12872_2020_1528_MOESM1_ESM.docx]

|  | **Hazard ratio** | **95% CI** | | **P value** |
| --- | --- | --- | --- | --- |
|  |  | **Lower** | **Upper** |  |
| **All-cause mortality at longest follow up** |  |  |  |  |
| 20-25 (Normal BMI) | Reference |  |  |  |
| <20 | 1.304 | 0.856 | 1.987 | 0.217 |
| 25-30 | 1.121 | 0.940 | 1.336 | 0.204 |
| 30-35 | 1.048 | 0.860 | 1.276 | 0.644 |
| >=35 | 1.238 | 0.986 | 1.555 | 0.066 |

Table S1
